# Synovial tissue‐derived extracellular vesicles induce chondrocyte inflammation and degradation via NF‐κB signalling pathway: An *in vitro* study

**DOI:** 10.1111/jcmm.17227

**Published:** 2022-02-18

**Authors:** Pu Chen, Jun Zhou, Anmin Ruan, Hua Guan, Jiewei Xie, Lingfeng Zeng, Jun Liu, Qingfu Wang

**Affiliations:** ^1^ Department of Orthopaedic Surgery Guangdong Provincial Hospital of Chinese Medicine (The 2nd Affiliated Hospital Guangzhou University of Chinese Medicine) Guangzhou China; ^2^ Department of Orthopaedic Surgery Beijing University of Chinese Medicine Third Affiliated Hospital Beijing China; ^3^ Department of Orthopaedic Surgery Beijing Longfu Hospital Beijing China; ^4^ 74715 Bone and Joint Research Team of Degeneration and Injury Guangdong Provincial Academy of Chinese Medical Sciences Guangzhou China; ^5^ Guangdong Second Traditional Chinese Medicine Hospital (Guangdong Province Engineering Technology Research Institute of Traditional Chinese Medicine) Guangzhou China

**Keywords:** cartilage degeneration, extracellular vesicles, NF‐κB signalling pathway, osteoarthritis, synovial inflammation

## Abstract

Osteoarthritis (OA) is a whole‐joint disease characterized by synovial inflammation and cartilage degeneration. However, the relationship between synovial inflammation and cartilage degeneration remains unclear. The modified Hulth's method was adopted to establish a knee OA (KOA) rabbit model. Synovial tissue was collected after 8 weeks, and synovial tissue‐derived extracellular vesicles (ST‐EVs) were extracted by filtration combined with size exclusion chromatography (SECF), followed by identification through transmission electron microscopy (TEM), nanoparticle tracer analysis (NTA) and Western blot (WB). The collagenase digestion method was used to extract normal rabbit chondrocytes, which were then treated with the SF‐EVs to observe the effect and mechanism of SF‐EVs on chondrocytes. The morphology, particle size and labelled protein marker detection confirmed that SECF successfully extract ST‐EVs. The ST‐EVs in the KOA state significantly inhibited chondrocyte proliferation and promoted chondrocytes apoptosis. Moreover, the ST‐EVs also promoted the expression of pro‐inflammatory cytokines (IL‐1β, IL‐6, TNF‐α and COX‐2) and cartilage degradation‐related enzymes (MMP13, MMP9 and ADAMTS5) in the chondrocytes. Mechanistically, the ST‐EVs significantly promoted the activation of NF‐κB signalling pathway in chondrocytes. Inhibition the activation of the NF‐κB signalling pathway significantly rescued the expression of inflammatory cytokines and cartilage degradation‐related enzymes in the ST‐EVs–induced chondrocytes. In conclusion, the ST‐EVs promote chondrocytes inflammation and degradation by activating the NF‐κB signalling pathway, providing novel insights into the occurrence and development of OA.

## 1 INTRODUCTION

Osteoarthritis (OA) is a common complex disease that manifests as pain, swelling and limited mobility.^1^, ^2^ Epidemiological studies have shown that OA affects more than 10% of the elderly,^3^ and 140 million people worldwide suffer from OA.^1^ To date, the pathogenesis of OA is not completely understood.^4^, ^5^ Cartilage degeneration has long been regarded to play a vital role in the pathogenesis of OA.^6^, ^7^, ^8^, ^9^ However, intensive research suggests that inflammation in the synovial tissue may play a pioneering role in the early stage of OA. Based on a case–control study, Atukorala et.al suggested that synovitis is a precursor of radiographic OA and increases the risk of cartilage loss.^10^ Wang et al. reported that synovial tissue may cause pathological changes in cartilage via the innate immune system to aggravate the progression of OA.^11^ However, how the inflammation of synovial tissue aggravates OA cartilage degeneration and how the signal between synovium and cartilage is transmitted remains unclear.

Extracellular vesicles (EVs), a type of microvesicle with a diameter of about 30–150 nm, a round or elliptical cup holder‐like structure, have a double‐layer phospholipid membrane structure, and play an important role in exchanging information between cells and tissues.^12^ EVs are widely present in cell medium supernatants and bodily fluids.^12^ Kato extracted exosomes from IL‐1β‐induced fibroblast‐like synoviocytes (FLS) supernatants, and then added FLS‐exosomes to chondrocytes. The results showed that FLS‐exosomes significantly promoted the expression of matrix metalloproteinase‐13 (MMP13) and decreased the expression of collagen II, thereby accelerating the cartilage degeneration.^13^ These results suggested that synovial tissue inflammation may regulate cartilage degeneration by secreting EVs. Several studies have investigated the extraction and mechanism of EVs in cell supernatants and body fluids; however, only a few articles have studied the extraction and mechanism of tissue‐derived EVs.

In our previous study,^14^ for the first time, we extracted and identified synovial tissue‐derived EVs (ST‐EVs), and found that the background of ST‐EVs extracted by the size exclusion + ultrafiltration method (SECF) was the cleanest, without the expression of EVs negative markers, and recommended for the follow‐up study of the function and mechanism of ST‐EVs. However, the effect and mechanism of ST‐EVs on OA cartilage are still unclear. Therefore, in this study, we used the modified Hulth's method to establish an OA rabbit model, which was used to extract ST‐EVs after 8 weeks, and then, the ST‐EVs were added to normal chondrocytes to observe their effect on OA state in cartilage and to study the mechanism underlying this effect.

## 2 METHODS

### 2.1 Reagents

For cell culture, Dulbecco's Modified Eagle Medium high‐glucose (DMEM‐H) were purchased from Hyclone (Boston, USA); antibiotic mixture (Penicillin and Streptomycin) was obtained from Invitrogen (CA, USA); exosome free foetal bovine serum was purchased from ABW (Uruguay). For Western blotting, primary antibodies against CD63, flotillin‐1, and calnexin were obtained from CST (USA); goat anti‐rabbit and goat anti‐mouse horseradish peroxidase conjugates were purchased from Proteintech (USA); BCA protein concentration assay kit was purchased from Thermo Scientific (USA). For RT‐PCR, RNA‐easy Isolation Reagent, PrimeScript Reverse Transcription kit and SYBR Premix Ex Taq II kit were purchased from Vazyme (Nanjing, China). Size exclusion chromatography and filtration kit was purchased from ECHO Biotech (Beijing, China). CCK‐8 assay kit was purchased from Dojindo (Tokyo, Japan). ELISA kit (IL‐1β, IL‐6, TNF‐α and COX2) was purchased from Meibiao Biotechnology (Jiangshu, China). Annexin‐V—FITC cell apoptosis detection kit was purchased from Solarbio (Beijing, China).

### 2.2 Establishment of OA rabbit model

Eight male rabbits (6 months, 2.5 ± 0.5 kg) were purchased from Beijing Keyu Animal Breeding Center (production license: SCXK (Jing)‐2018–0010), and then raised in separate cages in the Medical Animal Experiment Center of the Chinese Academy of Chinese Medical Sciences. The experimental protocols were approved by the Animal Care and Use Committee of China Academy of Chinese Medical Sciences. All rabbits are fed under the same breeding conditions, temperature: 21 ± 3℃, humidity: 55 ± 5%, dark/light cycle: 12/12 h. After one week of adaptive feeding, the modified Hulth's method was used to establish a moderate KOA rabbit model as previously described.^11^ Briefly, after anaesthesia, a 1 cm incision was made on the inside of the right knee joint, transected the medial collateral ligament (MCL) and then resection of the anterior cruciate ligament and medial meniscus. Penicillin was continuously injected for 3 days after the operation to prevent infection, and the rabbits were driven to simulate exercise for 30 min every day after modelling. After 8 weeks, the synovial tissue of the right knee was taken and extracted EVs by SECF; the cartilage of the right knee was taken for morphological detection, and the cartilage of left knee was taken and then digested into chondrocytes with collagenase.

### 2.3 Collection and treatment of synovial tissue specimens

As previously described,^14^ the obtained synovial tissue is immediately placed in PBS containing 1% antibiotic mixture, and transferred to the ultra‐clean table as soon as possible. After washed 3 times with PBS containing 1% antibiotic mixture, the synovial tissue was cut into pieces of 1 × 1 × 1 mm^3^, added the culture medium (containing 10% exosome free foetal bovine serum and 1% antibiotic mixture), and the culture supernatant was collected after 24 h. The collected medium was filtered through a 0.22 μm filter and SECF was used to extracted the ST‐EVs, all procedures were performed in strict accordance with the kit instructions.

### 2.4 Chondrocytes isolation

Primary normal rabbit chondrocytes were isolated from articular cartilage of left knee joint by collagenase digestion as previously described.^15^ In a nutshell, after washing with PBS containing 1% antibiotic mixture, the articular cartilage was cut into small pieces, and then digested with type II collagenase for 6 h. The filtered supernatant was centrifuged for 5 min, and the pellet was washed twice with ice‐cold PBS and resuspended in DMEM F12 with 10% FBS and 1% antibiotic mixture.

### 2.5 EVs characterization and uptake of EVs

As previously described,^14^ transmission electron microscopy (TEM) and nanoparticle tracking analysis (NTA) were performed to detect the morphology, particle size and concentration of EVs. For uptake of EVs, ST‐EVs were labelled with PKH67 (Sigma‐Aldrich) according to the manufacturer's protocol. Simply put, 1 × 10^7^ ST‐EVs were suspended in 1 ml PBS mixed with PKH67 dye, incubated at 4°C for 5 min, and then stopped the labelling reaction by the addition of BSA. The labelled EVs were incubated with primary normal rabbit chondrocytes for 4 h, and then visualized with fluorescence microscope.

### 2.6 Cell counting kit‐8 assay

Cell Counting Kit‐8 **(**CCK‐8**)** assay was used to assess the cell viability according to the manufacturer's protocol. Simply put, primary normal rabbit chondrocytes were cultured in 96‐well plates at a density of 8 × 10^3^ cells per well for 24 h and then pretreated with different concentrations of ST‐EVs for an additional 24 h. EVs concentration was defined as number of EVs per ml, which was based on the counting with the NTA. The supernatant of each group was aspirated, washed with PBS, 10 ml of CCK‐8 medium was added to each well and then incubated at 37°C for 3 h. The cell viability of each well was determined by measuring absorbance at 450 nm.

### 2.7 Western Blot (WB)

Chondrocytes were lysed RIPA supplemented with phenylmethanesulfonylfluoride (PMSF), and the protein concentration was quantified by the BCA protein assay kit. Extracted proteins were analysed by sodium dodecyl sulphate‐polyacrylamide gel electrophoresis; then, transferred to PVDF membrane as previously described.^14^, ^15^ After incubating 5% non‐fat dried milk at room temperature for 1 h, add primary antibody and incubate overnight at 4℃. After four washes in TBST, membranes were incubated with secondary antibodies for 1 h at room temperature. Then, the membranes were washed with TBST for 5 min × 8 times. Finally, ECL was added for the visualized of proteins according to the manufacturer's protocol.

### 2.8 Real‐time quantitative polymerase chain reaction analysis

Total RNA was extracted from chondrocytes using TRIzol reagent according to the manufacturer's instructions. The concentration of RNA samples was determined using the NanoDrop spectrophotometer, and then, the quality of RNA samples was checked by the 260 nm/280 nm ratios were checked. Complementary DNA was prepared using a commercially available kit under the following conditions: 15 min at 50°C and 5 s at 85°C. Real‐time quantitative polymerase chain reaction (RT‐ qPCR) was performed using a thermocycler and SYBR Premix Ex Taq II Kit under the following conditions: 10 min at 95°C, followed by 40 cycles at 95°C for 15 s and at 60°C for 1 min. The expression of GAPDH was used to normalize data, and the data were analysed using the 2^−ΔΔCT^ method. The RT‐qPCR primers are listed in Table 1.

**TABLE 1:** Primers sequences of the targeted genes

Primers	Sequences
IL−6‐F	GAGGCACTGGCGGAAGTCAATC
IL−6‐R	GAAGTGATTCTCAGCAGGCAGGTC
IL−1β‐F	ACCAACAAGTGGTGTTCTCCAT
IL−1β‐R	TGGGTAACGGTTGGGGTCTA
TNF‐α‐F	CATCTTCTCAAAATTCGAGTGACAA
TNF‐α‐R	TGGGAGTAGACAAGGTACAACCC
COX−2‐F	CACGCAGGTGGAGATGATCTAC
COX−2‐R	ACTTCCTGGCCCACAGCAAACT
MMP13‐F	TCTACACCTACACCGGCAAGAGTC
MMP13‐R	CGGAGACTGGTAATGGCATCAAGG
MMP9‐F	GTGAAGACGCAGACGGTGGATTC
MMP9‐R	GGTACTCACACGCCAGAAGAAGC
ADAMTS5‐F	TGGGCTACTCAGCCACAAAG
ADAMTS5‐R	CCACTGCAGCTGTGACGTAT
β‐Actin‐F	TGGCCGAGGACTTTGATTGT
β‐Actin‐R	ACACAAATGCGATGCTGCC

### 2.9 Flow cytometry

The chondrocytes of all groups were harvested. The cells were detached by EDTA‐free trypsin, collected by centrifuge with the supernatant removed, and then washed three times by pre‐cooled PBS at 4℃, centrifugation at 1000 rpm for 5 min. Removed the supernatant, resuspended the chondrocytes, added 2 μl mixture of Annexin‐V—FITC, PI and HEPES buffer to chondrocytes according to the instructions of Annexin‐V—PI cell apoptosis detection kit and incubated for 15 min on the ice in the dark. Then, the cell suspension was transferred to detection tubes, which were added with 300 μl PBS. Cell apoptosis was then analysed by the detection of FITC and PI fluorescence through the activation of bandpass at 525 nm and 620 nm and at a wavelength of 488 nm. Cell apoptosis rate = apoptotic cells / (apoptotic cells +normal cells).

### 2.10 Histological analysis

The femoral cartilage samples and synovial tissue were fixed in 4% paraformaldehyde, and then decalcified using the EDTA (Sigma‐Aldrich, US) microwave method. The cartilage samples were sectioned after conventional paraffin‐embedding. Put the cartilage sections in xylene Ⅰ and Ⅱ for 5 min each for dewaxing, and then debenzene step by step. Afterwards, haematoxylin and eosin (HE) staining was performed to reveal the cartilage morphology.

### 2.11 Enzyme‐linked immunosorbent assay

Briefly, chondrocyte supernatants were collected, and then, the proteins of the chondrocyte supernatants were extracted with RIPA and PMSF buffer. BCA protein assay kit was used to quantified the concentration of proteins. Afterwards, commercially available enzyme‐linked immunosorbent assay (ELISA) kit was used to detecte the expression of (IL‐1β, IL‐6, TNF‐a and COX2) according to the manufacturer's protocol.

### 2.12 Statistical analyses

In general, data obtained from this research were presented as the mean ± standard deviation (SD). All analyses were performed using GraphPad Prism 7.0 software. The statistical differences among groups were analysed by t test or one‐way ANOVA. *p* < 0.05 was considered statistically significant.

## 3 RESULTS

### 3.1 Isolation and identification of ST‐EVs

**FIGURE 1:**
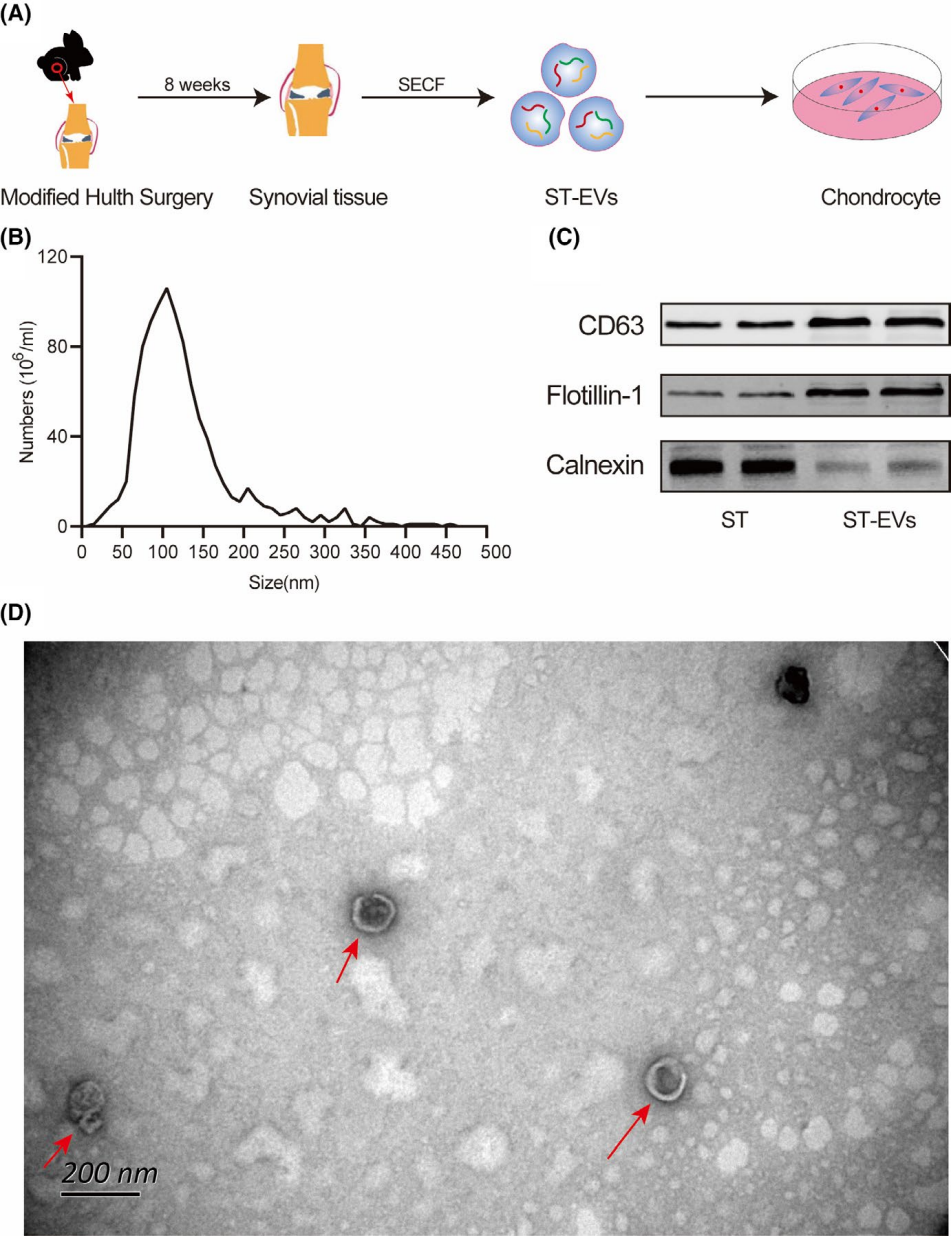
Isolation and identification of synovial tissue‐derived extracellular vesicles (ST‐EVs). (A) the flow chart of isolation of ST‐EVs; (B) size distribution of ST‐EVs measured by nanoparticle tracer analysis (NTA); (C) morphology of ST‐EVs observed by transmission electron microscopy (TEM); (D) surface markers of ST‐EVs measured by Western blot (WB)

To evaluate the effect and mechanism of ST‐EVs on OA cartilage, the modified Hulth's method was used to establish an OA rabbit model, to extract ST‐EVs after 8 weeks of modelling, and then interfere with normal chondrocytes (Figure 1A). NTA results showed that the particle size of ST‐EVs was mainly distributed in the range of 30–150 nm, and the main peak was approximately 100–120 nm, which is consistent with the particle size of exosomes (Figure 1B). Furthermore, the exosomal positive markers CD63 and flotillin‐1 were highly expressed in ST‐EVs, while the negative marker—calnexin was expressed at significantly lower level (Figure 1C). The TEM results showed that ST‐EVs had circular and elliptical disc‐shaped and cup holder‐shaped vesicles, about 30–150 nm in size, consistent with the morphological characteristics of EVs (Figure 1D).

### 3.2 OA model identification and EVs uptake by chondrocytes

**FIGURE 2:**
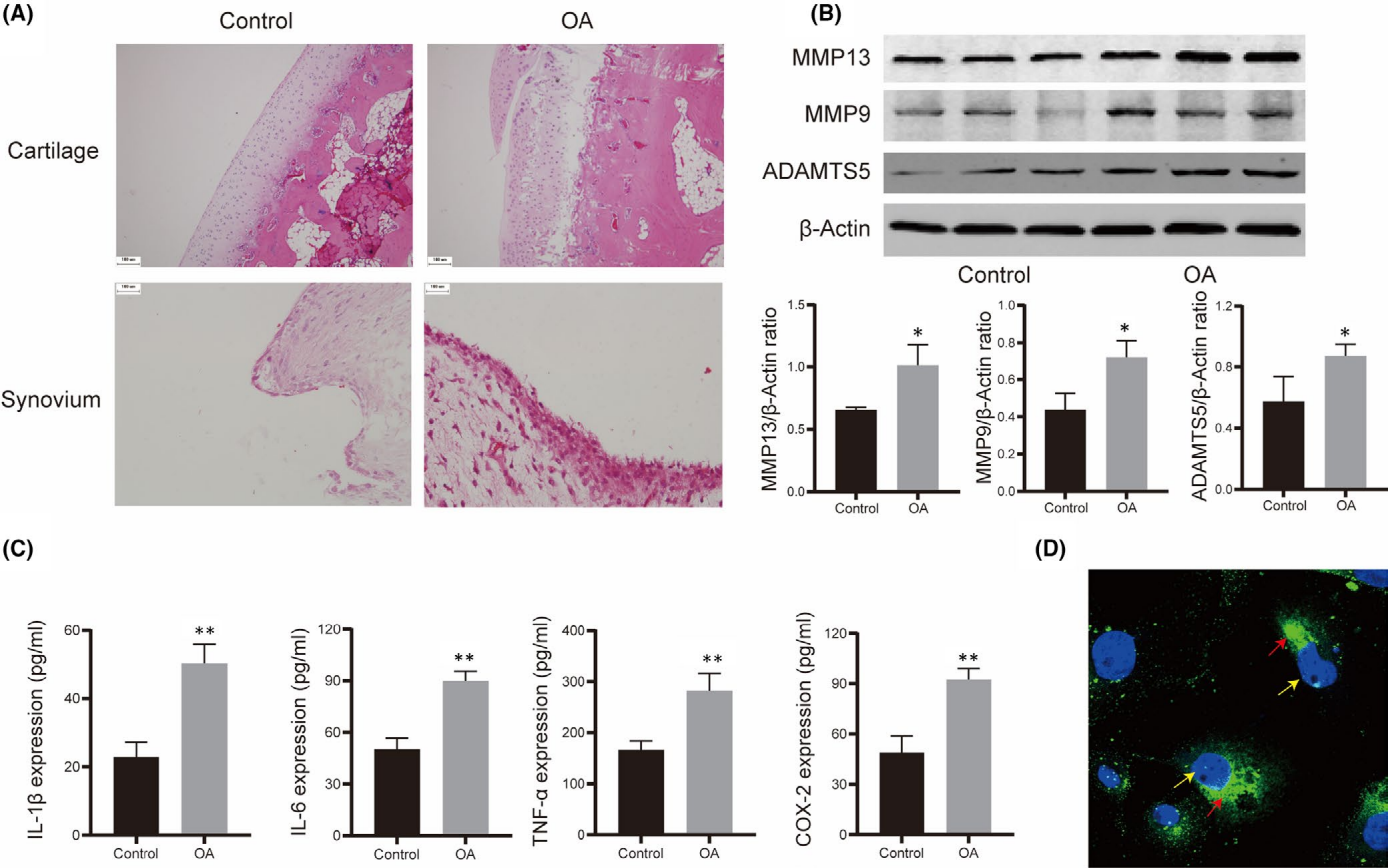
Osteoarthritis (OA) model identification and EVs uptake by chondrocytes. (A) HE stained sections of femoral cartilage samples and synovial tissue; (B) the expression of cartilage degradation‐related enzymes (MMP13, MMP9 and ADAMTS5) in cartilage measured by WB; (C) the expression of inflammatory cytokines (IL‐1β, IL‐6, TNF‐α and COX‐2) in synovial tissue detected by ELISA; (D) mode pattern of coculture of chondrocytes and ST‐EVs, the yellow arrow indicated chondrocytes, red arrow indicated PKH67‐labelled ST‐EVs. *N* = 3, *p < 0.05; **p < 0.01

To verify the effect of ST‐EVs in the KOA state on cartilage, we employed the modified Hulth's method to establish the OA rabbit model. HE staining of the femoral condyle cartilage showed a perfectly normal, physiological cartilage structure, with a smooth surface, regularly distributed cells and morphology with regular tidal lines. After 8 weeks, the surface of cartilage, distribution of chondrocytes and tidal line integrity in the OA model groups was gradually changed compared with the control group (Figure 2A). The cartilage surface was rough with slight cracks, increased cell number, cell clusters appeared and overlapping and disappearance of tidal lines (Figure 2A). Morphological changes in the synovial tissue were also observed. The normal synovial tissue structure is basement membrane‐free soft tissue arranged in a tile form. HE staining of the synovial tissue in the control group showed the synovial lining cells were distributed in a single or double‐layer structure without hyperplasia, hypertrophy, oedema or inflammatory infiltrate (Figure 2A). The structure of the synovial tissue in the OA group showed proliferation and accumulation of synovial lining cells, interstitial oedema, occasional inflammatory infiltrates and blood vessel proliferation (Figure 2A).

To further verify the reliability of the OA model, we examined the expression of cytokines in the cartilage and synovial tissues at the molecular level. The expression of cartilage degradation‐related enzymes (MMP13, MMP9 and ADAMTS5) in the cartilage was evaluated. As shown in Figure 2B, compared with the control group, the expression of cartilage degradation‐related enzymes in the cartilage was significantly higher in the OA group. In addition, we also evaluated the expression of inflammatory cytokines (IL‐1β, IL‐6, TNF‐α and COX‐2) in synovial tissue. The expression of the inflammatory cytokines was significantly higher in the OA group compared with the control group (Figure 2C). Morphological and molecular changes in the cartilage and synovial tissue suggested that the OA model was successfully established.

Furthermore, to observe that ST‐EVs can be absorbed by chondrocytes, green fluorescent (PKH67) labelled ST‐EVs were used to interfere with normal chondrocytes. As shown in Figure 2D, PKH67‐labelled ST‐EVs were localized in the perinuclear region of the chondrocytes, indicating that chondrocytes can internalize ST‐EVs.

### 3.3 ST‐EVs inhibit chondrocytes proliferation and promote apoptosis

**FIGURE 3:**
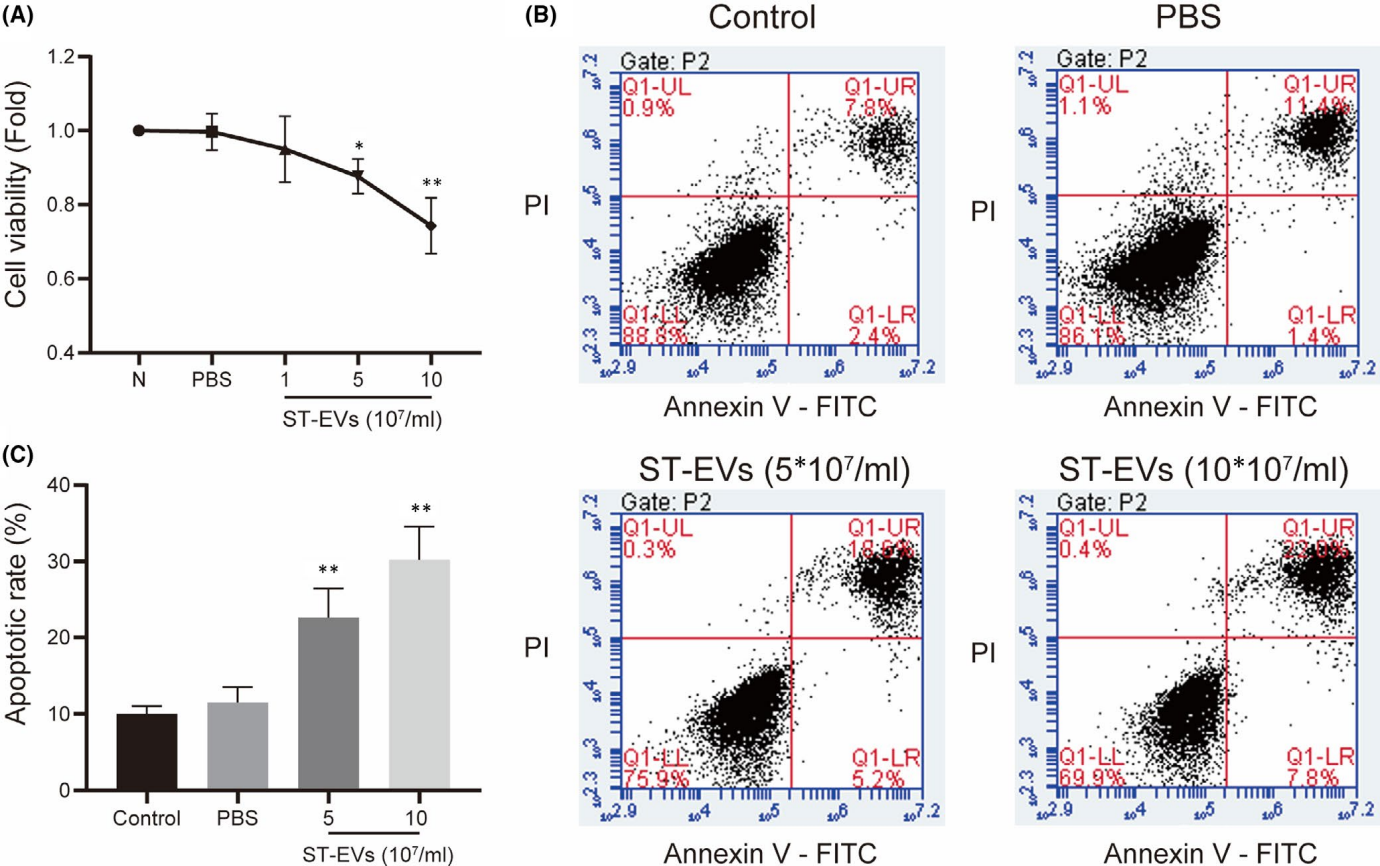
ST‐EVs inhibit the cell viability of chondrocytes and promote apoptosis. Normal chondrocytes were incubated with different concentrations of ST‐EVs (1, 5, 10 × 10^7^/ml) for 24 h. (A) the cell viability was evaluated using the CCK‐8 assay; (B,C) the apoptotic rate of chondrocytes was detected by Annexin‐V—PI Apoptosis Detection Kit and analysed with FlowJo software. *N* = 3, *p < 0.05; **p < 0.01

To detect the effect of ST‐EVs on the cell viability of chondrocytes, the CCK‐8 assay was used to detect the cell viability of chondrocytes. As shown in Figure 3A, following intervention with ST‐EVs, the cell viability of chondrocyte gradually decreased; while the ST‐EVs at a concentration of 5, 10 × 10^7^/ml, the cell viability of chondrocytes was significantly decreased (*p* < 0.05), and the difference increased with the concentration of ST‐EVs. Therefore, ST‐EVs at a concentration of 5, 10 × 10^7^/ml were used to interfere with chondrocytes in the subsequent experiments. In addition, we evaluated the effect of ST‐EVs on chondrocytes apoptosis (Figure 3B,C). The apoptotic rate of chondrocytes was significantly increased on treatment with ST‐EVs at a concentration of 5, 10 × 10^7^/ml. These results indicate that ST‐EVs in the KOA state significantly lowered the cell viability of chondrocytes and promoted chondrocytes apoptosis.

### 3.4 ST‐EVs promote the release of cartilage degradation‐related enzymes and inflammatory cytokines on chondrocytes

**FIGURE 4:**
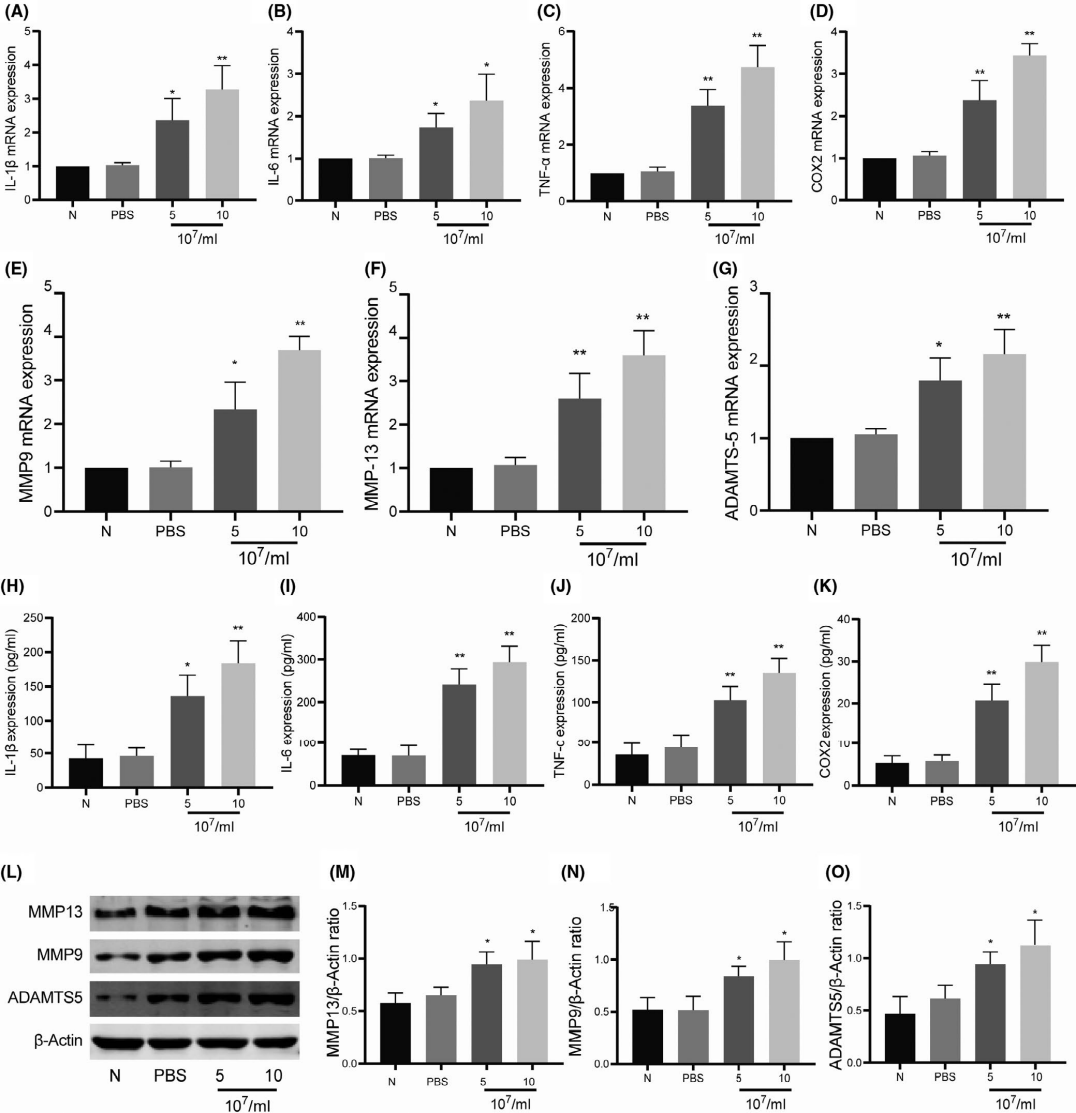
ST‐EVs promote the release of cartilage degradation‐related enzymes and inflammatory cytokines on chondrocytes. Normal chondrocytes were incubated with different concentrations of ST‐EVs (5, 10 × 10^7^/ml) for 24 h. (A‐D) the mRNA expression of inflammatory cytokines (IL‐1β, IL‐6, TNF‐α and COX‐2) in chondrocytes; (E‐G) the mRNA expression of cartilage degradation‐related enzymes (MMP13, MMP9 and ADAMTS5) in chondrocytes; (H‐K) the protein expression of inflammatory cytokines (IL‐1β, IL‐6, TNF‐α and COX‐2) in chondrocyte supernatants; L‐O, the protein expression of cartilage degradation‐related enzymes (MMP13, MMP9 and ADAMTS5) in chondrocytes. *N* = 3, *p < 0.05; **p < 0.01

Furthermore, we observed the effect of ST‐EVs on the inflammation and degeneration of normal cartilage in the KOA state. As shown in Figure 4A–G, compared with the control group, the mRNA expression of inflammatory cytokines (IL‐1β, IL‐6, TNF‐α and COX‐2) and cartilage degradation‐related enzymes (MMP13, MMP9 and ADAMTS5) was significantly increased after intervention with ST‐EVs in the KOA state at a concentration of 5, 10 × 10^7^/ml. Subsequently, ELISA and WB were used to detect the protein expression of inflammatory cytokines and cartilage degradation‐related enzymes in the chondrocytes. As shown in Figure 4H–O, changes in the protein expression of inflammatory cytokines and cartilage degradation‐related enzymes paralleled changes in mRNA expression. The ST‐EVs under KOA state significantly promoted inflammation and degeneration of normal chondrocytes.

### 3.5 ST‐EVs promote the inflammation and degeneration of normal cartilage via NF‐κB signalling pathway

**FIGURE 5:**
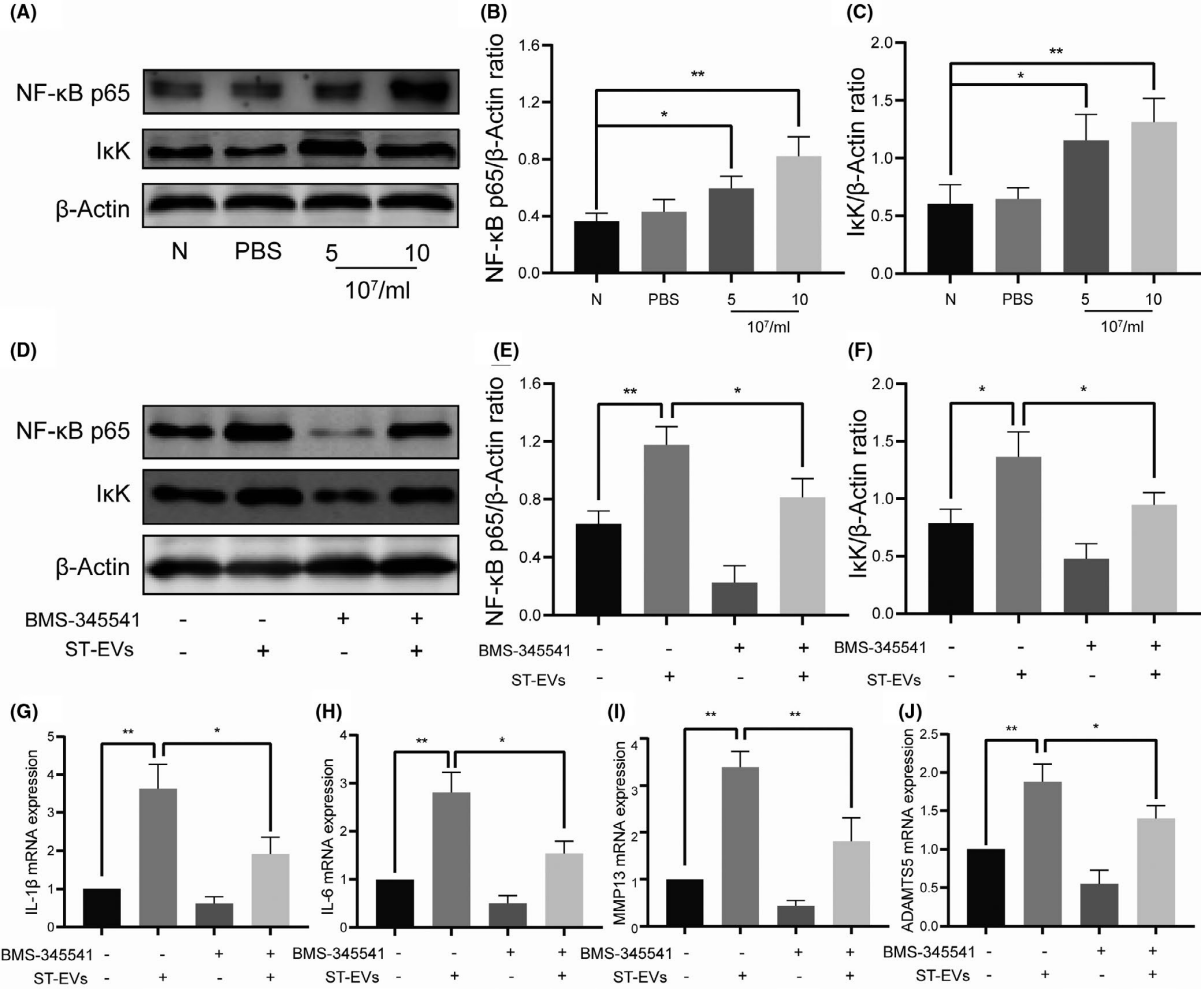
ST‐EVs promote cartilage inflammation and degeneration via NF‐κB signalling pathway. Normal chondrocytes were incubated with different concentrations of ST‐EVs (5, 10 × 10^7^/ml) for 24 h, and the expression of NF‐κB p65 and IκK in chondrocytes was detected by WB (A‐C). Normal chondrocytes were incubated with ST‐EVs for 24 h after pretreatment with or without BMS‐345541 (5 μM), WB was used to detect the expression of NF‐κB p65 and IκK in chondrocytes (D‐F), and PCR was used to detect the expression of IL‐1β, IL‐6, MMP13 and ADAMTS5 in chondrocytes (G‐J). *N* = 3, *p < 0.05; **p < 0.01

The NF‐κB signalling pathway is widely involved in the regulation of immune and inflammatory responses, cell proliferation, tumorigenesis and other physiological processes. It has been previously reported that NF‐κB signalling pathway plays an important role in mediating cartilage degeneration and inflammation in OA. Therefore, we hypothesized that the NF‐κB signalling pathway might play a role in ST‐EVs‐induced cartilage degeneration and inflammation. As shown in Figure 5A–C, strong activation of the NF‐κB signalling pathway was observed in the chondrocytes in response to ST‐EVs (10 × 10^7^/ml) intervention. Subsequently, we assessed the role of the NF‐κB signalling pathway in ST‐EVs‐mediated cartilage inflammation and damage. As shown in Figure 5D–F, ST‐EVs (10 × 10^7^/ml) intervention in chondrocytes significantly activated the NF‐κB signalling pathway, whereas BMS‐345541, an inhibitor of the NF‐κB signalling pathway, significantly reversed the activation of the NF‐κB signalling by the ST‐EVs. Furthermore, we evaluated the effect of inhibiting the NF‐κB signalling pathway on ST‐EVs in promoting cartilage damage and inflammation. As shown in Figure 5G–J, inhibition of the NF‐κB signalling significantly reduced the expression of IL‐1β, IL‐6, MMP13 and ADAMTS5 in ST‐EVs‐induced chondrocytes. These results indicate that the ST‐EVs promote the inflammation and degeneration of normal chondrocytes by activating the NF‐κB signalling pathway.

## 4 DISCUSSION

KOA is a common condition affecting middle‐aged and older individuals. It is driven by synovial inflammation, progressive cartilage degradation, osteophyte formation and subchondral bone sclerosis.^1^, ^16^ Although many methods are known for the treatment of KOA, currently there are no effective treatments to reverse the pathological changes during the occurrence and development of KOA. Total knee arthroplasty is considered to be the golden method in the treatment of end‐stage KOA patients.^17^ It has a significant risk of complications including revision, infection and unsatisfactory function, with high traumatic and high treatment cost.^17^, ^18^, ^19^ Therefore, effectively delaying the progression of KOA and avoiding the premature progression of early and mid‐stage KOA to end‐stage KOA has been a focus of orthopaedic research.

Synovial inflammation is widespread during the progression of KOA and remains one of the most important pathological features of KOA. Many studies have shown a correlation between synovial inflammation and KOA symptoms. Baker et al.^20^ conducted a multicentre cohort study using enhanced magnetic resonance imaging (CE‐MRI) in 535 participants to assess the correlation between synovial inflammation and pain. The results showed that synovial inflammation is closely related to the severity of pain and Kellgren–Lawrence grade, and the correlation between synovial inflammation and pain is persistent in KOA patients without imaging findings. Guermazi et al. evaluated synovial inflammation semi‐quantitatively based on CE‐MRI, and assessed the relationship between KOA synovitis and cartilage damage and meniscus damage in 404 patients. They found that among patients whose X‐ray examination revealed KOA, the higher the Kellgren–Lawrence grade, the higher the probability of synovitis; the higher the synovitis score, the higher the degree of cartilage damage.^21^ Atukorala et al.^10^ followed up for up to 8 years for people without KOA imaging findings and found that among patients with KOA imaging findings during the follow‐up period, synovial inflammation first appeared with cartilage damage, further suggesting that synovium during the KOA process inflammation may affect cartilage damage. However, the mechanism by which the inflammation of synovial tissue exacerbates cartilage degeneration, and how the signal is transmitted between synovial tissue and cartilage tissue is still unclear.

Under normal physiological conditions, the synovial membrane releases a lubricant and hyaluronic acid, which play important roles in reducing joint surface friction, protecting the cartilage surface, and maintaining cartilage homeostasis.^22^, ^23^ When stimulated, the synovium can secrete a large number of inflammatory cytokines, such as TNF‐α, IL‐1β, IL‐6, IL‐8, IL‐17 and PGE2, which can cause irreversible cartilage degeneration and aggravate the progression of KOA.^23^, ^24^, ^25^, ^26^, ^27^ Samavedi et al. co‐cultured chondrocytes and synovial macrophages in an inflammatory state and found that the β‐catenin signalling pathway in chondrocytes was significantly activated, secreted the inflammatory cytokines, such as TNF‐α, IL‐1β, and IL‐6, increased the expression of extracellular matrix degradation‐related enzymes, such as MMP1, MMP3, MMP9 and MMP13, which promote cartilage degeneration.^28^ This was also nicely shown in cocultures of tissue explants of cartilage and synovium.^29^ Kato et al. enriched the FLS‐derived exosomes in the KOA state, and then interfered with normal chondrocytes.^13^ The results showed that the expression of collagen Ⅱ and extracellular matrix degradation‐related enzymes such as MMP13 and ADAMTS5 was significantly increased in chondrocytes. Meanwhile, Zhou et al.^30^ found that FLS‐derived exosomes overexpressing miR‐126‐3p significantly inhibited chondrocyte inflammation and delayed chondrocyte degeneration, further suggesting that synovial cells may affect chondrocytes by secreting exosomes. However, the current research on the effect of synovial cells on chondrocytes is limited to a certain type of synovial cells. The function of ST‐EVs and its effect on chondrocytes has not yet been studied.

**FIGURE 6:**
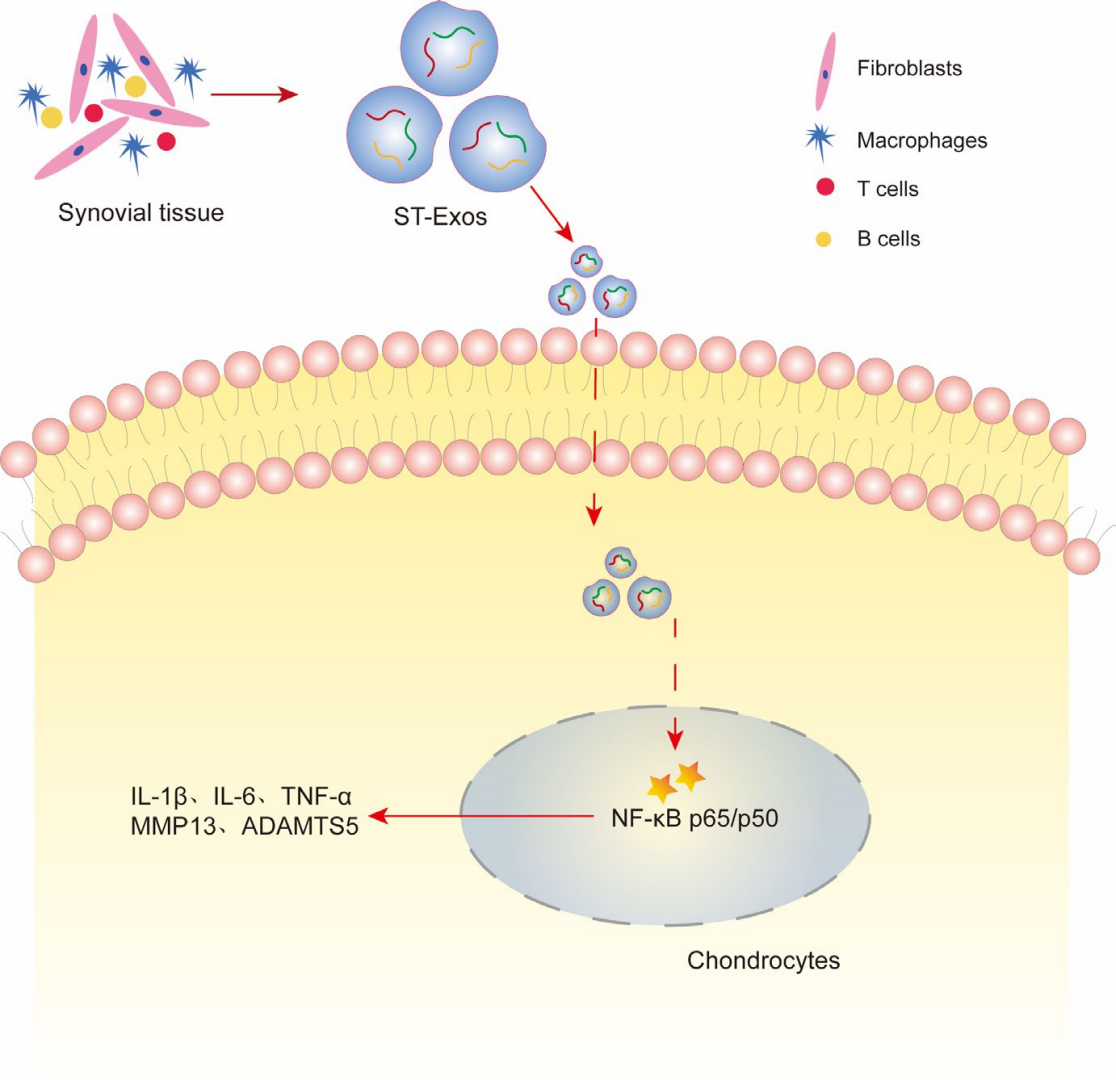
Schematic presentation of ST‐EVs mediated cartilage inflammation and degeneration under KOA state

In a previously published research,^14^ we used ultracentrifugation (UC), filtration combined with size exclusion chromatography (SECF) and 8% polyethylene glycol (PEG) to extract ST‐EVs, and found that the background of EVs extracted by the SECF method was the cleanest, with no negative marker protein expression. Therefore, in this study, we used SECF to extract ST‐EVs. Similarly, morphology, particle size and labelled protein marker detection confirmed that SECF can successfully extract ST‐EVs. Subsequently, we evaluated the effect of ST‐EVs on chondrocytes and found that as the concentration of ST‐EVs increased, the activity of chondrocytes gradually decreased. Meanwhile, we observed the effect of ST‐EVs on cartilage inflammation and degeneration in the KOA state, and found that ST‐EVs significantly promoted the expression of inflammatory cytokines (IL‐1β, IL‐6, TNF‐α and COX‐2) and cartilage degradation‐related enzymes (MMP13, MMP9 and ADAMTS5), suggesting that ST‐EVs promote cartilage inflammation and degeneration. To explore the mechanism of action of ST‐EVs on cartilage inflammation and degeneration, we observed the expression of the NF‐κB signalling pathway in chondrocytes. In addition, we found that the NF‐κB signalling pathway was significantly activated in chondrocytes after ST‐EVs intervention, and inhibition of NF‐κB signalling pathway significantly reversed the effect of ST‐EVs on cartilage inflammation and degeneration. These findings suggest that ST‐EVs promote cartilage inflammation and degradation via activating the NF‐κB signalling pathway (Figure 6).

While our results provide clear evidence that synovial inflammation in KOA state can promote cartilage inflammation and degeneration through EVs, there are certain limitations to this study. How the ST‐EVs activate the NF‐κB signalling pathway in chondrocytes, and then aggravate cartilage degeneration remains unclear. Meanwhile, it is also unclear whether there is a difference in the contents of ST‐EVs between the normal state and KOA states. To better understand the complexities of the KOA pathological process, in our next study, we plan to explore which genes are wrapped in ST‐EVs to activate the NF‐κB signalling pathway in chondrocytes and promote cartilage inflammation and degeneration.

In summary, our findings indicate that synovial inflammation can significantly promote cartilage damage and chondrocyte inflammation and degradation via activating the NF‐κB signalling pathway, which provides novel insight into the occurrence and development of OA (Figure 6).

## CONFLICT OF INTEREST

The authors declare that they have no conflict of interest.

## AUTHOR CONTRIBUTIONS

**Pu Chen:** Conceptualization (equal); Data curation (equal); Validation (equal); Visualization (equal); Writing – original draft (equal); Writing – review & editing (equal). **Jun Zhou:** Investigation (equal); Validation (equal); Visualization (equal). **Anmin Ruan:** Data curation (equal); Validation (equal); Visualization (equal). **Hua Guan:** Methodology (equal); Software (equal); Visualization (equal). **Jiewei Xie:** Methodology (equal); Resources (equal); Validation (equal). **Lingfeng Zeng:** Resources (equal); Software (equal). **Jun Liu:** Conceptualization (equal); Funding acquisition (equal); Supervision (equal). **QingFu Wang:** Conceptualization (equal); Funding acquisition (equal); Supervision (equal).

## Data Availability

All reagents used in this work are available on request and a brief statement describing the purpose for their use.

## References

[1] Kloppenburg M , Berenbaum F . Osteoarthritis year in review 2019: epidemiology and therapy. Osteoarthritis Cartilage. 2020;28(3):242‐248. doi:10.1016/j.joca.2020.01.002 31945457

[2] Hunter DJ , Bierma‐Zeinstra S . Osteoarthritis. Lancet. 2019;393(10182):1745‐1759. doi:10.1016/s0140-6736(19)30417-9 31034380

[3] Han B , Li Q , Wang C , et al. Decorin regulates the aggrecan network integrity and biomechanical functions of cartilage extracellular matrix. ACS Nano. 2019;13(10):11320‐11333. doi:10.1021/acsnano.9b04477 31550133 PMC6892632

[4] Son YO , Kim HE , Choi WS , Chun CH , Chun JS . RNA‐binding protein ZFP36L1 regulates osteoarthritis by modulating members of the heat shock protein 70 family. Nat Commun. 2019;10(1):77. doi:10.1038/s41467-018-08035-7 30622281 PMC6325149

[5] Klein JC , Keith A , Rice SJ , et al. Functional testing of thousands of osteoarthritis‐associated variants for regulatory activity. Nature Commun. 2019;10(1):2434. doi:10.1038/s41467-019-10439-y 31164647 PMC6547687

[6] Konttinen YT , Sillat T , Barreto G , Ainola M , Nordstrom DC . Osteoarthritis as an autoinflammatory disease caused by chondrocyte‐mediated inflammatory responses. Arthritis Rheum. 2012;64(3):613‐616. doi:10.1002/art.33451 22130805

[7] Sacchetti C , Liu‐Bryan R , Magrini A , Rosato N , Bottini N , Bottini M . Polyethylene‐glycol‐modified single‐walled carbon nanotubes for intra‐articular delivery to chondrocytes. ACS Nano. 2014;8(12):12280‐12291. doi:10.1021/nn504537b 25415768 PMC4373402

[8] Wang Q , Rozelle AL , Lepus CM , et al. Identification of a central role for complement in osteoarthritis. Nat Med. 2011;17(12):1674‐1679. doi:10.1038/nm.2543 22057346 PMC3257059

[9] Amiable N , Tat SK , Lajeunesse D , et al. Proteinase‐activated receptor (PAR)‐2 activation impacts bone resorptive properties of human osteoarthritic subchondral bone osteoblasts. Bone. 2009;44(6):1143‐1150. doi:10.1016/j.bone.2009.02.015 19264156 PMC5250314

[10] Atukorala I , Kwoh CK , Guermazi A , et al. Synovitis in knee osteoarthritis: a precursor of disease? Ann Rheum Dis. 2016;75(2):390‐395. doi:10.1136/annrheumdis-2014-205894 25488799 PMC4916836

[11] Wang H , Wang Q , Yang M , et al. Histomorphology and innate immunity during the progression of osteoarthritis: does synovitis affect cartilage degradation? J Cell Physiol. 2018;233(2):1342‐1358. doi:10.1002/jcp.26011 28513840

[12] Thery C , Witwer KW , Aikawa E , et al. Minimal information for studies of extracellular vesicles 2018 (MISEV2018): a position statement of the International Society for Extracellular Vesicles and update of the MISEV2014 guidelines. J Extracell Vesicles. 2018;7(1):1535750. doi:10.1080/20013078.2018.1535750 30637094 PMC6322352

[13] Kato T , Miyaki S , Ishitobi H , et al. Exosomes from IL‐1beta stimulated synovial fibroblasts induce osteoarthritic changes in articular chondrocytes. Arthrit Res Ther. 2014;16(4):R163. doi:10.1186/ar4679 PMC4261911 25092378

[14] Chen PU , Ruan A , Zhou J , et al. Extraction and identification of synovial tissue‐derived exosomes by different separation techniques. J Orthop Surg Res. 2020;15(1):97. doi:10.1186/s13018-020-01604-x 32151262 PMC7063768

[15] Chen PU , Ruan A , Zhou J , et al. Cinnamic aldehyde inhibits lipopolysaccharide‐induced chondrocyte inflammation and reduces cartilage degeneration by blocking the nuclear factor‐kappa B signaling pathway. Front Pharmacol. 2020;11:949. doi:10.3389/fphar.2020.00949 32848721 PMC7419651

[16] Glyn‐Jones S , Palmer AJ , Agricola R , et al. Osteoarthritis. Lancet. 2015;386(9991):376‐387. doi:10.1016/S0140-6736(14)60802-3 25748615

[17] Jevsevar DS , Brown GA , Jones DL , et al. The American Academy of Orthopaedic Surgeons evidence‐based guideline on: treatment of osteoarthritis of the knee, 2nd edition. J Bone Joint Surg Am. 2013;95(20):1885‐1886. doi:10.2106/00004623-201310160-00010 24288804

[18] Hossain F , Patel S , Haddad FS . Midterm assessment of causes and results of revision total knee arthroplasty. Clin Orthop Relat Res. 2010;468(5):1221‐1228. doi:10.1007/s11999-009-1204-0 20058112 PMC2853653

[19] D'Apuzzo M , Westrich G , Hidaka C , Jung Pan T , Lyman S . All‐cause versus complication‐specific readmission following total knee arthroplasty. J Bone Joint Surg Am. 2017;99(13):1093‐1103. doi:10.2106/jbjs.16.00874 28678122 PMC5490331

[20] Baker K , Grainger A , Niu J , et al. Relation of synovitis to knee pain using contrast‐enhanced MRIs. Ann Rheum Dis. 2010;69(10):1779‐1783. doi:10.1136/ard.2009.121426 20472593 PMC3885343

[21] Guermazi A , Hayashi D , Roemer FW , et al. Synovitis in knee osteoarthritis assessed by contrast‐enhanced magnetic resonance imaging (MRI) is associated with radiographic tibiofemoral osteoarthritis and MRI‐detected widespread cartilage damage: the MOST study. J Rheumatol. 2014;41(3):501‐508. doi:10.3899/jrheum.130541 24429179 PMC5476295

[22] Hui AY , McCarty WJ , Masuda K , Firestein GS , Sah RL . A systems biology approach to synovial joint lubrication in health, injury, and disease. Wiley Interdiscip Rev Syst Biol Med. 2012;4(1):15‐37. doi:10.1002/wsbm.157 21826801 PMC3593048

[23] Smith MD . The normal synovium. Open Rheumatol J. 2011;5:100‐106. doi:10.2174/1874312901105010100 22279508 PMC3263506

[24] Macchi V , Stocco E , Stecco C , et al. The infrapatellar fat pad and the synovial membrane: an anatomo‐functional unit. J Anat. 2018;233(2):146‐154. doi:10.1111/joa.12820 29761471 PMC6036933

[25] Mathiessen A , Conaghan PG . Synovitis in osteoarthritis: current understanding with therapeutic implications. Arthrit Res Ther. 2017;19(1):18. 10.1186/s13075-017-1229-9 PMC5289060 28148295

[26] Kennedy A , Fearon U , Veale DJ , Godson C . Macrophages in synovial inflammation. Front Immunol. 2011;2:52. doi:10.3389/fimmu.2011.00052 22566842 PMC3342259

[27] Bhattaram P , Chandrasekharan U . The joint synovium: a critical determinant of articular cartilage fate in inflammatory joint diseases. Semin Cell Dev Biol. 2017;62:86‐93. doi:10.1016/j.semcdb.2016.05.009 27212252

[28] Zhang H , Lin C , Zeng C , et al. Synovial macrophage M1 polarisation exacerbates experimental osteoarthritis partially through R‐spondin‐2. Ann Rheum Dis. 2018;77(10):1524‐1534. doi:10.1136/annrheumdis-2018-213450 29991473

[29] Beekhuizen M , Bastiaansen‐Jenniskens YM , Koevoet W , et al. Osteoarthritic synovial tissue inhibition of proteoglycan production in human osteoarthritic knee cartilage: establishment and characterization of a long‐term cartilage‐synovium coculture. Arthritis Rheum. 2011;63(7):1918‐1927. doi:10.1002/art.30364 21437876

[30] Zhou Y , Ming J , Li Y , et al. Exosomes derived from miR‐126‐3p‐overexpressing synovial fibroblasts suppress chondrocyte inflammation and cartilage degradation in a rat model of osteoarthritis. Cell Death Discov. 2021;7(1):37. doi:10.1038/s41420-021-00418-y 33627637 PMC7904758

